# *CDHR1* variants in a Japanese family with inherited retinal dystrophy and intrafamilial phenotypic variability

**DOI:** 10.3389/fopht.2026.1736277

**Published:** 2026-02-06

**Authors:** Toshiaki Hirakata, Dan Gao, Minami Oshima, Fumihiro Hara, Shintaro Nakao, Akira Murakami

**Affiliations:** Department of Ophthalmology, Juntendo University Graduate School of Medicine, Tokyo, Japan

**Keywords:** case series, CDHR1, inherited retinal dystrophy, intrafamilial variability, rod–cone dystrophy, variant of uncertain significance

## Abstract

**Introduction:**

To report a Japanese family with inherited retinal dystrophy (IRD) in which *CDHR1* variants were identified, and to characterize the marked intrafamilial phenotypic variability.

**Methods:**

This retrospective case series included three brothers diagnosed with retinal dystrophy at Juntendo University Hospital. Comprehensive ophthalmic evaluations were performed, including best-corrected visual acuity (BCVA), Goldmann perimetry, fundus photography, fundus autofluorescence (FAF), optical coherence tomography (OCT), and full-field electroretinogram (ERG). Genetic testing was conducted using next-generation sequencing with an IRD gene panel.

**Results:**

All three patients exhibited progressive visual decline with onset in their 40s–50s. Fundus examination revealed severe macular atrophy in two brothers (Cases 1 and 2), consistent with cone–rod dystrophy, whereas the youngest (Case 3) showed diffuse retinal degeneration with bone-spicule pigmentation resembling retinitis pigmentosa. FAF demonstrated hypoautofluorescence in the macula and hyperautofluorescence at the borders of atrophic areas in Cases 1 and 2, but widespread hypoautofluorescence in Case 3. ERG revealed rod–cone dysfunction in Cases 1 and 2 and non-recordable responses in Case 3. Genetic analysis identified a single heterozygous *CDHR1* c.748C>A (p.Pro250Thr) variant in Case 1. In Cases 2 and 3, two heterozygous *CDHR1* variants—c.748C>A (p.Pro250Thr) and c.2027T>A (p.Ile676Asn)—were detected. Case 1 as having a single heterozygous *CDHR1* variant with a phenotype overlapping that of Cases 2 and 3, and explicitly note that the genetic diagnosis in Case 1 remains inconclusive.

**Conclusions:**

This study describes a Japanese family with IRD showing substantial intrafamilial phenotypic heterogeneity, ranging from macular-predominant cone–rod dystrophy to generalized rod–cone dystrophy, in the context of identified *CDHR1* variants. These findings highlight the complexity of genotype–phenotype correlations in *CDHR1*-related retinal disease and underscore the importance of cautious interpretation of genetic results, particularly when variants of uncertain significance are identified.

## Introduction

Autosomal recessive inherited retinal dystrophies (IRDs) are genetically heterogeneous and often associated with loss of function of cone and rod photoreceptors, potentially leading to blindness (1). *Cadherin-related family member 1* (*CDHR1*) is a causative gene for retinal dystrophy, with an autosomal recessive form of inheritance (2, 3). The cadherin-related family member 1 (CDHR1), also known as protocadherin 21, or photoreceptor-specific cadherin, is a structural transmembrane photoreceptor protein that localizes at the base of cone and rod photoreceptor outer segments and plays an essential role in the maintenance of the structure and survival of photoreceptors (4). It is encoded by *CDHR1*, a gene located on chromosome 10q23.1 (5).

*Cdhr1*-deficient mice exhibit progressive outer retinal degeneration and early electroretinographic abnormalities involving both rod and cone pathways.

The *Cdhr1*-deficient mouse exhibits structural and functional evidence of progressive outer retinal degeneration at a slow rate (6). And, electroretinograms (ERGs) showed early functional deficits affecting both rod and cone photoreceptors. In humans, *CDHR1* variants have been associated with a range of phenotypes, including cone–rod dystrophies (CRD) (7, 8), rod–cone dystrophies (RCD), retinitis pigmentosa (RP) (9), and late-onset macular degeneration (LOMD) (10, 11).

In this study, we report a Japanese family harboring a novel *CDHR1* variant and exhibiting marked intrafamilial phenotypic variability, highlighting the complexity of genotype–phenotype correlations in *CDHR1*-associated retinal dystrophies and the importance of comprehensive clinical and genetic evaluation.

## Methods

This was a retrospective case series of patients presenting to Juntendo University hospital with a diagnosis of IRDs. Three male siblings from the same Japanese family were included. Detailed medical history and comprehensive ophthalmic examinations were performed, including visual acuity, visual field test, fundus photography (CLARUS 500; Carl Zeiss Meditec Inc., Dublin, CA, USA), fundus autofluorescence (CLARUS 500; Carl Zeiss Meditec Inc., Dublin, CA, USA), optical coherence tomography (OCT, Spectralis; Heidelberg Engineering, Inc., Heidelberg, Germany), full-field electroretinography (full-field ERG, LE-4000, Tomey, Nagoya, Japan), were performed in accordance with ISCEV standards (12). Genetic analysis was conducted using next-generation system (NGS) with a gene panel. Pathogenicity of identified variants was interpreted with reference to established databases and ACMG guidelines. All *CDHR1* variants were annotated based on the RefSeq transcript NM_033100.4. This study was conducted in accordance with the Declaration of Helsinki and approved by the Institutional Review Board of Juntendo University (M08-0468). Written informed consent was obtained from all participants.

## Results

### Clinical characteristics

The family comprises four siblings, with the eldest being a sister followed by three younger brothers, of whom the three brothers were included in this study. Case 1 was a Japanese male in his late 60s who first noted night blindness in his early 50s. His past ocular history included laser *in situ* keratomileusis (LASIK) at the age 52 and bilateral cataract surgery at the age 60.

Case 2 was a Japanese male in his mid-60s who developed night blindness in his late 40s, followed by photophobia in his early 50s. His medical history included hypertension managed with medication, and prior surgeries for hemorrhoids and lumbar disc herniation. He was a former smoker, with a 15-cigarettes-per-day habit until age 57. His children were asymptomatic at the time of evaluation.

Case 3 was a Japanese male in his early 60s who experienced the onset of night blindness in his mid-40s, particularly under low-illumination or rainy conditions. He subsequently noted gradual progressive visual decline. He underwent bilateral cataract surgery in his late 50s, with the left eye procedure performed during an acute glaucoma episode.

**Figure 1:**
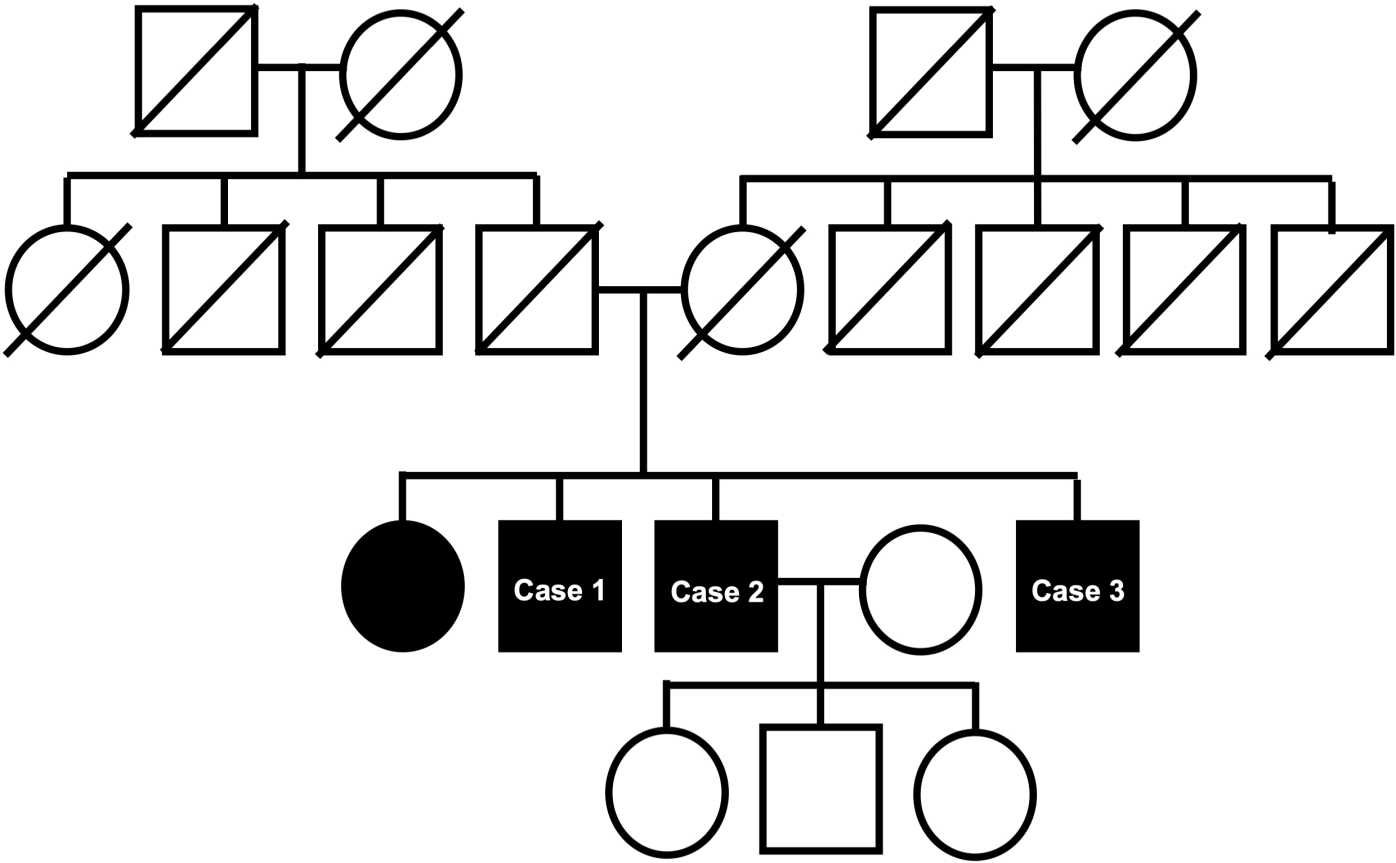
Pedigree of the family with haplotype bars. Filled symbols represent affected individuals, and open symbols indicate unaffected individuals.

The three brothers also have a sister who was diagnosed with IRD at another medical institution. Her symptoms began around age 50, and she currently has severe visual impairment and has been unable to renew her driver’s license since that time. The pedigree is shown in **Figure 1**.

### Vision and visual field

**Figure 2:**
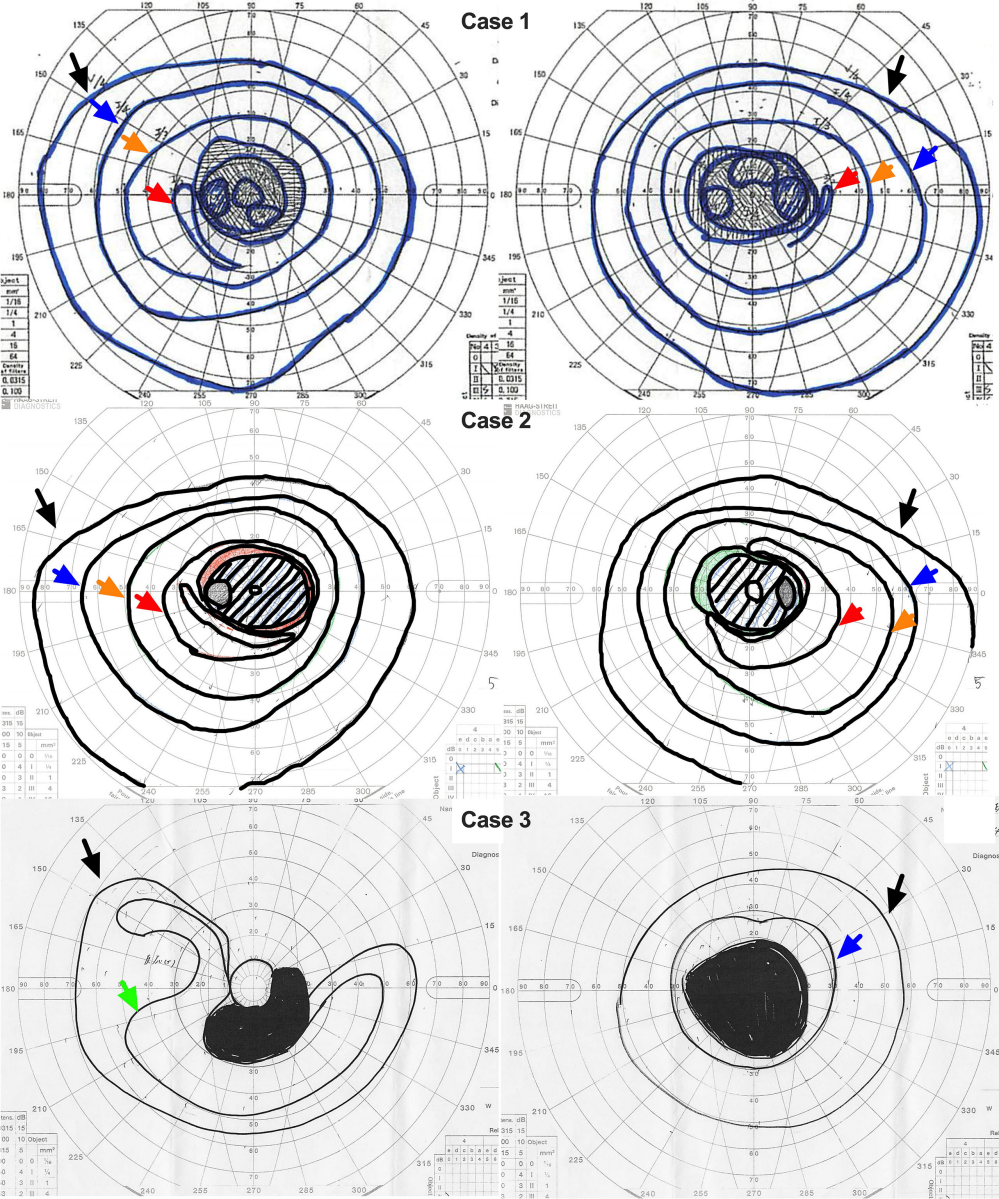
Goldmann perimetry (GP) findings in all three cases. GP results for Case 1, Case 2, and Case 3 are shown from top to bottom. The right and left columns correspond to the right and left eyes, respectively. Cases 1 and 2 demonstrated central scotomas in both eyes, whereas Case 3 exhibited a central scotoma accompanied by peripheral visual field constriction. Black arrows indicate the visual field delineated by the V/4 isopter; a green arrow, the III/4 isopter; orange arrows, the I/3 isopter; blue arrows, the I/4 isopter; and red arrows, the I/2 isopter.

Goldmann perimetry (GP) of three cases was shown in **Figure 2**. At the initial visit, Case 1 (in his late 60s) had BCVA of 0.05 in the right eye and 0.06 in the left eye. GP revealed a central scotoma in both eyes.

At the initial visit, Case 2 (in his late 50s) had decimal visual acuities of 1.2 in the right eye and 0.9 in the left eye. His vision gradually deteriorated over the following several years, reaching approximately 0.1 or less in both eyes by his early 60s. GP demonstrated a central scotoma in both eyes, similar to Case 1.

At the initial visit, Case 3 (aged 61 years) had visual acuities of 0.4 in the right eye and 0.05 in the left eye. GP revealed peripheral visual field constriction and generalized narrowing of the visual field.

### Fundus images

**Figure 3:**
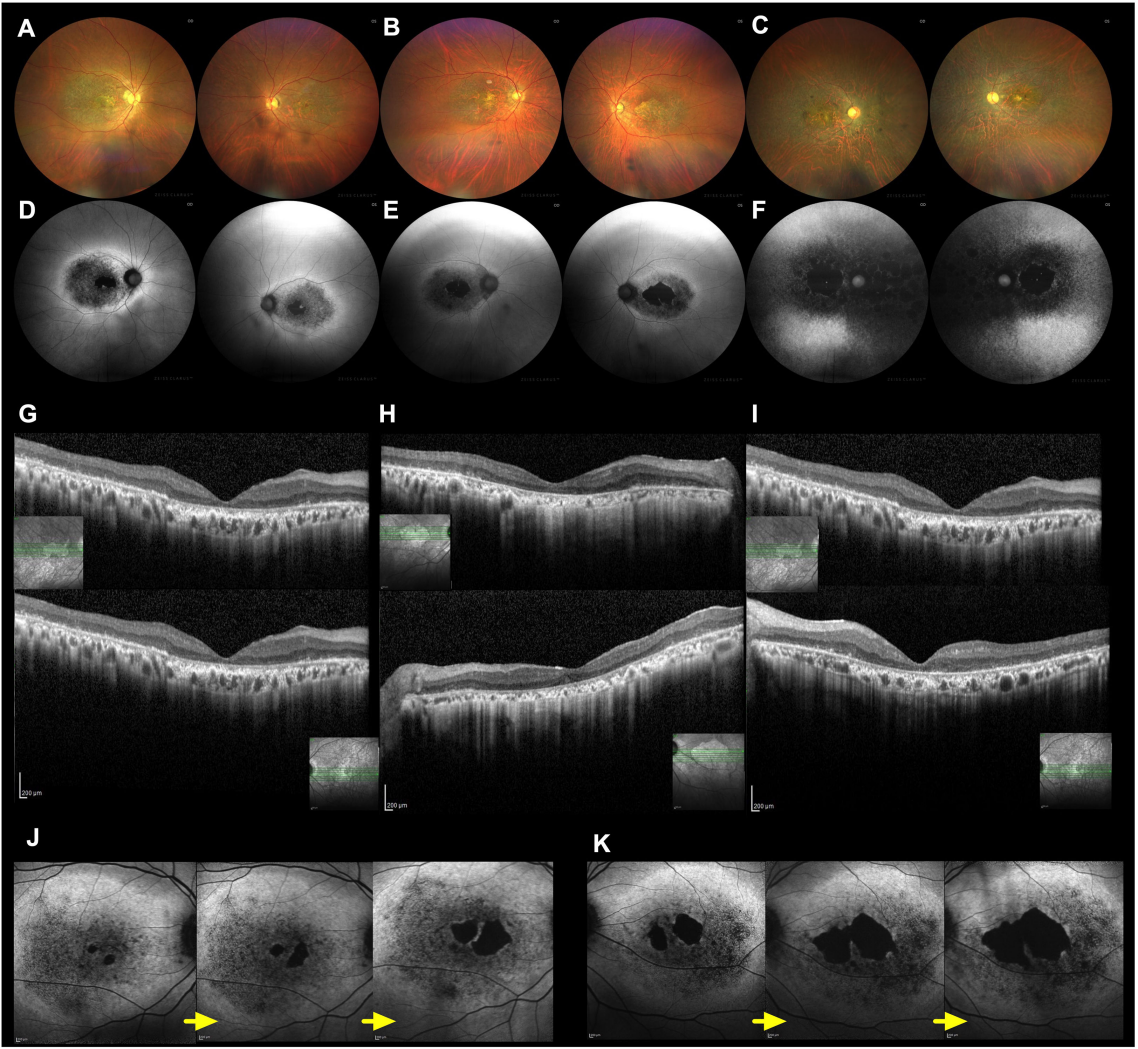
**(A−C)** shows the fundus photography of Cases 1, 2, and 3, respectively. **(D−F)** shows the fundus autofluorescence (FAF) images of Cases 1, 2, and 3, respectively. These images show macular degeneration and macular atrophy in both case 1 and 2, while not only macular atrophy and peripheral retinal degeneration in case 3. **(G−I)** Optical coherence tomography (OCT) images of Cases 1, 2, and 3, respectively (upper panels: right eyes; lower panels: left eyes). OCT images reveal outer retinal atrophy involving the retinal pigment epithelium (RPE) in the macular region of all three cases. **(J, K)** Longitudinal FAF images of Case 2 at ages 57, 58, and 61 years for the right and left eyes, respectively. In Case 2, the fovea was initially preserved; however, the hypoautofluorescent area corresponding to RPE atrophy gradually expanded over time.

Fundus photographs showed macular atrophy in both eyes of Cases 1 and 2, whereas in Case 3, retinal atrophy extended beyond the macula into the peripheral retina, accompanied by faint white spots and bone-spicule–like pigmentation in both eyes (**Figures 3A–C**). FAF demonstrated hypoautofluorescence in the macular area and surrounding hyperautofluorescence near the vascular arcades in both eyes of Cases 1 and 2 (**Figures 3D, E**). In contrast, FAF in Case 3 showed widespread hypoautofluorescence involving both the macula and peripheral retina (**Figure 3F**). OCT revealed loss of the outer retinal layers in the macula in all three cases (**Figures 3G–I**). Longitudinal FAF imaging clearly illustrated progressive macular degeneration in Case 2 (**Figures 3J, K**). Initially, visual acuity was preserved because the foveal retina remained intact; however, as foveal photoreceptor and retinal pigment epithelium (RPE) degeneration advanced, visual acuity gradually declined.

### Full-field electroretinogram

**Figure 4:**
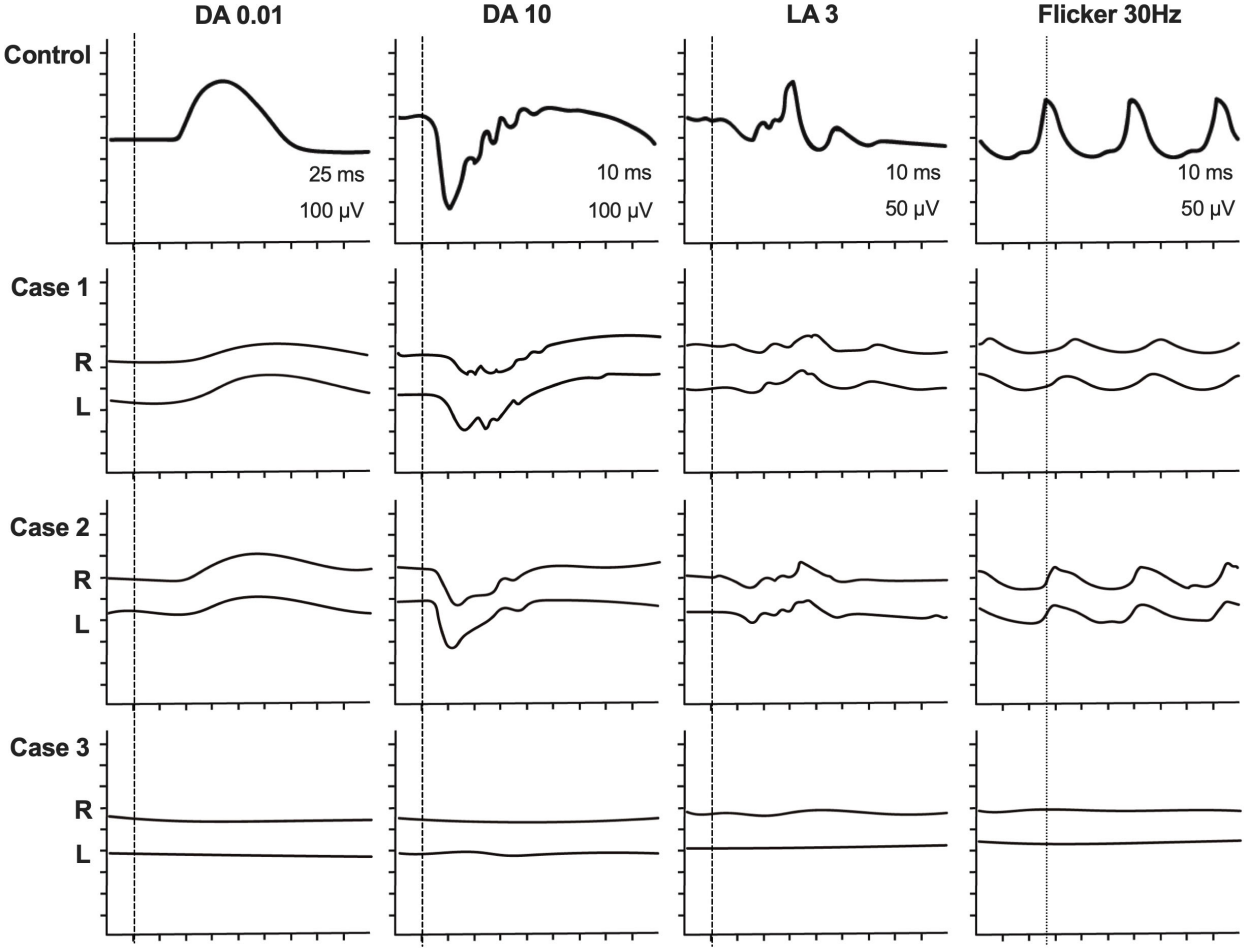
Full-field electroretinograms (ERGs) were recorded in all three cases. ERGs in the top row were shown as a normal control. In both case 1 and 2. Dark adapted dim flash ERG (0.01 cd•s•m-2; DA 0.01) showed a reduced response and bright flash ERG (DA 10) had severely reduced a-wave and b-wave. Light adapted cone ERG (LA 3) severely affected and 30Hz flicker ERG (LA 3, 30Hz) showed a reduction of amplitude and delayed implicate time. On the other hand, in case 3 full-field ERG showed un-detectable responses in both dark and light adapted conditions. Full-field electroretinograms (full-field ERGs) recorded in all three cases compared with a normal control (top row). In Cases 1 and 2, the dark-adapted dim-flash ERG (0.01 cd·s·m^-^²; DA 0.01) showed markedly reduced amplitudes, and the dark-adapted bright-flash ERG (DA 10) revealed severely attenuated a- and b-waves. The light-adapted single-flash ERG (LA 3) showed severely reduced cone responses, and the 30 Hz flicker ERG (LA 3, 30 Hz) demonstrated reduced amplitude and delayed implicit time. In contrast, Case 3 showed non-recordable responses under both dark- and light-adapted conditions.

Full-field ERG demonstrated combined rod and cone dysfunction in both Cases 1 and 2. The dark-adapted dim flash ERG (0.01 cd·s·m^-^²; DA 0.01) showed markedly reduced responses, and the dark-adapted bright-flash ERG (DA 10) revealed severely attenuated a- and b-waves. The light-adapted cone ERG (LA 3) showed a markedly reduced amplitude, and the 30-Hz flicker ERG (LA 3, 30 Hz) exhibited both decreased amplitude and delayed implicit time. In contrast, Case 3 showed non-recordable responses under both dark- and light-adapted conditions, indicating advanced retinal dysfunction (**Figure 4**).

### Gene analysis

The summary of gene analysis is shown in **Table 1**. Following the evaluation of the sequencing data, we identified a heterozygous variant *CDHR1* c.748C>A (p. Pro250Thr) in Case 1. Because only a single *CDHR1* allele was affected, the molecular diagnosis in Case 1 remains inconclusive with respect to autosomal recessive *CDHR1*-associated IRD. In Cases 2 and 3, two heterozygous *CDHR1* variants—c.748C>A (p.Pro250Thr) and c.2027T>A (p.Ile676Asn)—were detected. Because segregation analysis and phasing were not performed, the allelic configuration (cis or trans) could not be definitively determined, and therefore these variants are described as two heterozygous variants.

**Table 1:** Summary of gene analysis.

	Gene	Nucleotide change (HGVS)	Zygosity	rsID	ClinVar dbSNP rsID	ACMG	ToMMo 61KJPN(# of homozygous individuals)	gnomAD v.4.1.0	REVEL score	HGMD(Phenotype)	PMID
Case 1	*CDHR1*	c.748C>A p.(Pro250Thr)	heterozygous	−	−	VUS (PM2)	Not found	Not found	0.415	none	none
Case 2	*PROM1*	c.1738A>C p.(Asn580His)	heterozygous	rs199674847	Conflicting classifications of pathogenicity Uncertain significance(5); Benign(1); Likely benign(1)(★)	VUS (BS1)	0.007491(5)	Total:0.0001190 East Asian:0.004210	0.306	CM157898(Cone dystrophy)	PMID:26161267 PMID:31213501
	*CDHR1*	c.748C>A p.(Pro250Thr)	heterozygous	−	−	VUS (PM2)	Not found	Not found	0.415	none	none
		c.2027T>A p.(Ile676Asn)	heterozygous	rs186486854	Uncertain significance(★★)	VUS (BS1)	0.002372(2)	Total:0.00008178 East Asian:0.002809(homozygotes:1)	0.565	CM141772(Retinitis pigmentosa )?	PMID:24154662 PMID:30992995 PMID:33691693 PMID:33946315 PMID:33608557
Case 3	*PROM1*	c.1738A>C p.(Asn580His)	heterozygous	rs199674847	Conflicting classifications of pathogenicity Uncertain significance(5); Benign(1); Likely benign(1)(★)	VUS (BS1)	0.007491(5)	Total:0.0001190 East Asian:0.004210	0.306	CM157898(Cone dystrophy)	PMID:26161267 PMID:31213501
	*RP1*	c.392G>A p.(Arg131Gln)	heterozygous	rs752150870	Uncertain significance(★★)	VUS (BS1)	0.003173(1)	Total:0.00005771 East Asian:0.002052	0.375	none	PMID:31213501 PMID:34073704
	*CDHR1*	c.748C>A p.(Pro250Thr)	heterozygous	−	−	VUS (PM2)	Not found	Not found	0.415	none	none
		c.2027T>A p.(Ile676Asn)	heterozygous	rs186486854	Uncertain significance(★★)	VUS (BS1)	0.002372(2)	Total:0.00008178 East Asian:0.002809(homozygotes:1)	0.565	CM141772(Retinitis pigmentosa )?	PMID:24154662 PMID:30992995 PMID:33691693 PMID:33946315 PMID:33608557

The *CDHR1* c.748C>A (p.Pro250Thr) variant represents a novel variant that has not been previously reported. In addition, two variants of uncertain significance (VUS) were detected: a heterozygous *PROM1* c.1738A>C (p.Asn580His) variant in Cases 2 and 3, and a heterozygous *RP1* c.392G>A (p.Arg131Gln) variant in Case 3.

## Discussion

We report three brothers with IRD in whom *CDHR1* variants were identified, exhibiting marked phenotypic variability within the same family. Although the affected siblings showed overlapping clinical features, their genetic findings were not identical, and their clinical presentations differed substantially. Cases 1 and 2 showed similar findings characterized by severe macular atrophy, decreased visual acuity, and central scotoma, consistent with LOMD. However, the presence of night blindness and electroretinographic evidence of combined rod–cone dysfunction was atypical for isolated macular dystrophy. In contrast, Case 3 presented with fundus features resembling RP, including widespread retinal degeneration, bone-spicule pigmentation, subjective night blindness, and non-recordable full-field ERG responses, suggesting a rod–cone dystrophy phenotype.

Both *CDHR1* variants identified in this study are currently classified as VUS according to ACMG/AMP guidelines. The c.2027T>A (p.Ile676Asn) variant was initially assigned PP3 based on in silico prediction; however, upon re-evaluation, the REVEL score (0.565) does not meet the threshold recommended by the ClinGen Sequence Variant Interpretation Working Group for supporting pathogenicity. Therefore, PP3 should not be applied. This variant shows relatively higher allele frequencies in East Asian populations, including the Japanese cohort, with reported homozygous individuals in public databases, supporting the application of BS1. Nevertheless, in Cases 2 and 3, c.2027T>A was detected together with a rare *CDHR1* variant, c.748C>A (p.Pro250Thr), which is absent from population databases. Although parental segregation analysis was not available, the recurrent identification of these two heterozygous variants in affected siblings suggests a possible trans configuration. In this context, and consistent with the Japanese guideline of IRDs (13), PM3 supporting may be cautiously considered. Importantly, the available evidence remains insufficient to reclassify either variant as pathogenic or likely pathogenic, and both variants should be interpreted as VUS. Their contribution to disease pathogenesis remains hypothetical.

Case 1 carries only a single heterozygous *CDHR1* c.748C>A (p.Pro250Thr) variant.

This monoallelic VUS is insufficient to establish *CDHR1*-associated autosomal recessive disease. Alternative explanations include an undetected second pathogenic allele (e.g., deep intronic variant, regulatory variant, or copy-number variant) or other genetic contributors not captured by our panel-based NGS. Therefore, Case 1 is considered genetically inconclusive despite its clinical resemblance to Cases 2 and 3.

In addition, beyond the REVEL score shown in **Table 1**, no additional in silico prediction tools were applied in this study. Given the absence of segregation analysis and comprehensive in silico evidence, the pathogenic relevance of the identified *CDHR1* variants cannot be definitively established and should be interpreted with caution.

Variants in *CDHR1* have been previously associated with a spectrum of autosomal recessive retinal dystrophies, including CRD (7, 8); RCD and RP (9, 14), and more recently, LOMD (10, 11). Intrafamilial phenotypic heterogeneity among individuals carrying identical *CDHR1* variants has also been documented (7, 15). However, the mechanisms underlying these phenotypic differences remain poorly understood.

All three cases in this study shared several features: severe macular atrophy, late-onset symptoms, and self-reported night blindness, with ERG confirming dysfunction of both photoreceptor systems. These findings suggest that *CDHR1*-associated retinal dystrophy may present along a phenotypic continuum ranging from macular-predominant cone–rod dystrophy to generalized rod–cone dystrophy. Even in patients initially diagnosed with macular dystrophy, careful longitudinal follow-up is warranted to monitor for potential peripheral retinal involvement as the disease progresses.

An additional *CDHR1* variant, c.2027T>A (p.Ile676Asn), was identified in Cases 2 and 3; however, this variant is currently classified as VUS, and its pathogenic role remains unclear. Other variants identified in this family, including those in *PROM1* and *RP1*, may be relevant to retinal disease in general, but there is no direct evidence that they contribute to the observed phenotypic variability in the present cases.

Although non-Mendelian mechanisms such as genetic modifiers, oligogenic inheritance, or allelic expression imbalance have been proposed in IRD (16), these mechanisms remain speculative in the present family and are not supported by direct genetic or functional evidence. Accordingly, the phenotypic variability observed in this family should be interpreted with caution, and the possibility of additional undetected genetic or environmental factors cannot be excluded.

Although segregation analysis and functional validation were not performed, these findings expand the understanding of *CDHR1* genotype–phenotype correlations. We propose that *CDHR1* variants can manifest across a broad clinical spectrum, and that modifier variants or transcript-level regulation may underlie intrafamilial phenotypic diversity. Clinicians should consider *CDHR1* variants in patients with macular dystrophy accompanied by night blindness or ERG evidence of rod–cone dysfunction.

This study has several limitations, including the absence of segregation analysis in the parents and the lack of *in vitro* or *in vivo* functional studies. Nevertheless, our findings contribute valuable insights into the expanding genotype–phenotype correlations in *CDHR1*-related retinal dystrophy.

Furthermore, even in cases with fundus findings suggestive of macular dystrophy, it is crucial to consider *CDHR1* gene variants in clinical management when patients report night blindness or show evidence of rod-cone dysfunction on ERG. Taken together, our findings suggest that *CDHR1*-associated retinal dystrophy may manifest along a phenotypic continuum, ranging from macular-predominant disease to generalized retinal degeneration. However, the interpretation of genotype–phenotype correlations must be approached cautiously, particularly in the absence of segregation analysis and functional validation.

This study has several limitations, including the lack of segregation analysis, comprehensive structural variant detection, and *in vitro* or *in vivo* functional studies. Further investigations incorporating these approaches will be necessary to clarify the pathogenic relevance of the identified variants and to better understand the mechanisms underlying intrafamilial phenotypic variability in *CDHR1*-associated retinal dystrophy.

In conclusion, our findings illustrate marked intrafamilial phenotypic variability in a Japanese family with IRD in which *CDHR1* variants were identified. These observations highlight the importance of integrating molecular, electrophysiological, and imaging analyses, as well as careful interpretation of genetic findings, for accurate diagnosis, prognostic assessment, and genetic counseling of patients with IRDs.

## Data Availability

The datasets presented in this study can be found in online repositories. The names of the repository/repositories and accession number(s) can be found in the article/supplementary material.
